# Supplementary material for: Rethinking the Estrogen Receptor Beta Dominance Hypothesis in Endometriosis: Insights from Single Cell RNA Sequencing Meta-analysis
Source: bioRxiv. 2025 Sep 18:2025.09.15.676330. Preprint. [Version 1] doi: 10.1101/2025.09.15.676330 (PMC12458327; doi:10.1101/2025.09.15.676330)
Supplement: 1 [file NIHPP2025.09.15.676330V1-supplement-1.pdf]

bioRxiv preprint doi: https://doi.org/10.1101/2025.09.15.676330; this version posted September 18, 2025. The copyright holder for this preprint (which was not certified by peer review) is the author/funder, who has granted bioRxiv a license to display the preprint in perpetuity. It is made available under aCC-BY 4.0 International license.

Supplementary Table 1

Final Cell Counts Split by Tissue and Cell Types

	Stromal	Epithelial	Endothelial	Myeloid	Lymphocyte
Control Eutopic (CEuE)	79490	30654	13075	3719	21148
Disease Eutopic (DEuE)	81513	14751	12239	5272	20325
Ectopic Lesions (EcE)	59302	2989	6565	10150	45934
Disease Free Peritoneum (NED)	14570	953	1533	3043	6269
Ovarian Endometrioma (EcO)	26557	695	3710	3617	12498
Control Ovarian Tissue (COv)	13803	0	843	117	1444
Disease Ovarian Tissue (DOv)	9512	0	540	5002	4709
Control Peritoneal Fluid (CPF)	0	181	0	5390	1099
Disease Peritoneal Fluid (DPF)	0	169	0	6638	1821
Control Whole Menstrual Effluent (CWME)	937	325	0	1728	5795
Disease Whole Menstrual Effluent (DWME)	900	152	0	941	3850
Control Menstrual Effluent Tissue (CMET)	1259	778	0	564	1954
Disease Menstrual Effluent Tissue (DMET)	1367	286	63	751	3572

Supplementary Table 1: Final filtered cell counts derived from the count matrix for each tissue and cell type subgroup.

bioRxiv preprint doi: https://doi.org/10.1101/2025.09.15.676330; this version posted September 18, 2025. The copyright holder for this preprint (which was not certified by peer review) is the author/funder, who has granted bioRxiv a license to display the preprint in perpetuity. It is made available under aCC-BY 4.0 International license.

Supplementary Table 2

Final Cell Counts Split by Estrogen Receptor Isoform Expression					
	ESR1+	ESR2+	Both	Null	Total
Control Eutopic (CEuE)	73210	3199	4006	67671	148086
Disease Eutopic (DEuE)	52232	1048	1015	79805	134100
Ectopic Lesions (EcE)	12373	2699	364	109504	124940
Disease Free Peritoneum (NED)	3606	292	19	22451	26368
Ovarian Endometrioma (EcO)	2811	2126	161	41979	47077
Control Ovarian Tissue (COv)	827	55	3	15322	16207
Disease Ovarian Tissue (DOv)	372	153	10	19228	19763
Control Peritoneal Fluid (CPF)	916	75	8	5671	6670
Disease Peritoneal Fluid (DPF)	865	53	8	7702	8628
Control Whole Menstrual Effluent (CWME)	293	42	1	8449	8785
Disease Whole Menstrual Effluent (DWME)	124	27	0	5692	5843
Control Menstrual Effluent Tissue (CMET)	213	28	1	4313	4555
Disease Menstrual Effluent Tissue (DMET)	347	92	1	5599	6039
Total:	148189	9889	5597	393386	557061

Supplementary Table 2: Final filtered cell counts derived from the count matrix split by estrogen receptor isoform expression.

bioRxiv preprint doi: https://doi.org/10.1101/2025.09.15.676330; this version posted September 18, 2025. The copyright holder for this preprint (which was not certified by peer review) is the author/funder, who has granted bioRxiv a license to display the preprint in perpetuity. It is made available under aCC-BY 4.0 International license.

Supplementary Table 3

Wilcoxon Rank Sum P Values from ESR1 to ESR2 Comparisons in Each Subgroup

	Stromal	Epithelial	Endothelial	Myeloid	Lymphocyte
Control Eutopic (CEuE)	<2.2e-16	<2.2e-16	5.02e-15	<2.2e-16	<2.2e-16
Disease Eutopic (DEuE)	<2.2e-16	<2.2e-16	5.439e-15	<2.2e-16	<2.2e-16
Ectopic Lesions (EcE)	<2.2e-16	<2.2e-16	<2.2e-16	3.672e-10	<2.2e-16
Disease Free Peritoneum (NED)	<2.2e-16	<2.2e-16	1.656e-11	3.667e-15	0.0003387
Ovarian Endometrioma (EcO)	<2.2e-16	0.2768	<2.2e-16	0.07207	<2.2e-16
Control Ovarian Tissue (COv)	<2.2e-16	NA	0.0178	0.3142	1.962e-06
Disease Ovarian Tissue (DOv)	2.128e-09	NA	0.1792	3.278e-10	7.896e-06
Control Peritoneal Fluid (CPF)	NA	0.5256	NA	<2.2e-16	0.002244
Disease Peritoneal Fluid (DPF)	NA	0.04713	NA	<2.2e-16	0.02089
Control Whole Menstrual Effluent (CWME)	<2.2e-16	0.006243	NA	5.816e-11	2.25e-05
Disease Whole Menstrual Effluent (DWME)	<2.2e-16	0.0001847	NA	0.03219	0.0001412
Control Menstrual Effluent Tissue (CMET)	<2.2e-16	2.825e-11	NA	0.02027	0.002167
Disease Menstrual Effluent Tissue (DMET)	<2.2e-16	2.022e-13	0.3251	0.002611	2.948e-11

Supplementary Table 3: All p-values from Wilcoxon Rank Sum tests. We compared the distribution of raw counts of ESR1 to the distribution of raw counts of ESR2. A significant p value indicating that the two distributions are likely to be significantly different from one another. In most cases, ESR1 appeared to visually have higher counts than ESR2, but this did vary, particularly in the EcO tissue.

bioRxiv preprint doi: https://doi.org/10.1101/2025.09.15.676330; this version posted September 18, 2025. The copyright holder for this preprint (which was not certified by peer review) is the author/funder, who has granted bioRxiv a license to display the preprint in perpetuity. It is made available under aCC-BY 4.0 International license.

Supplementary Figure 1

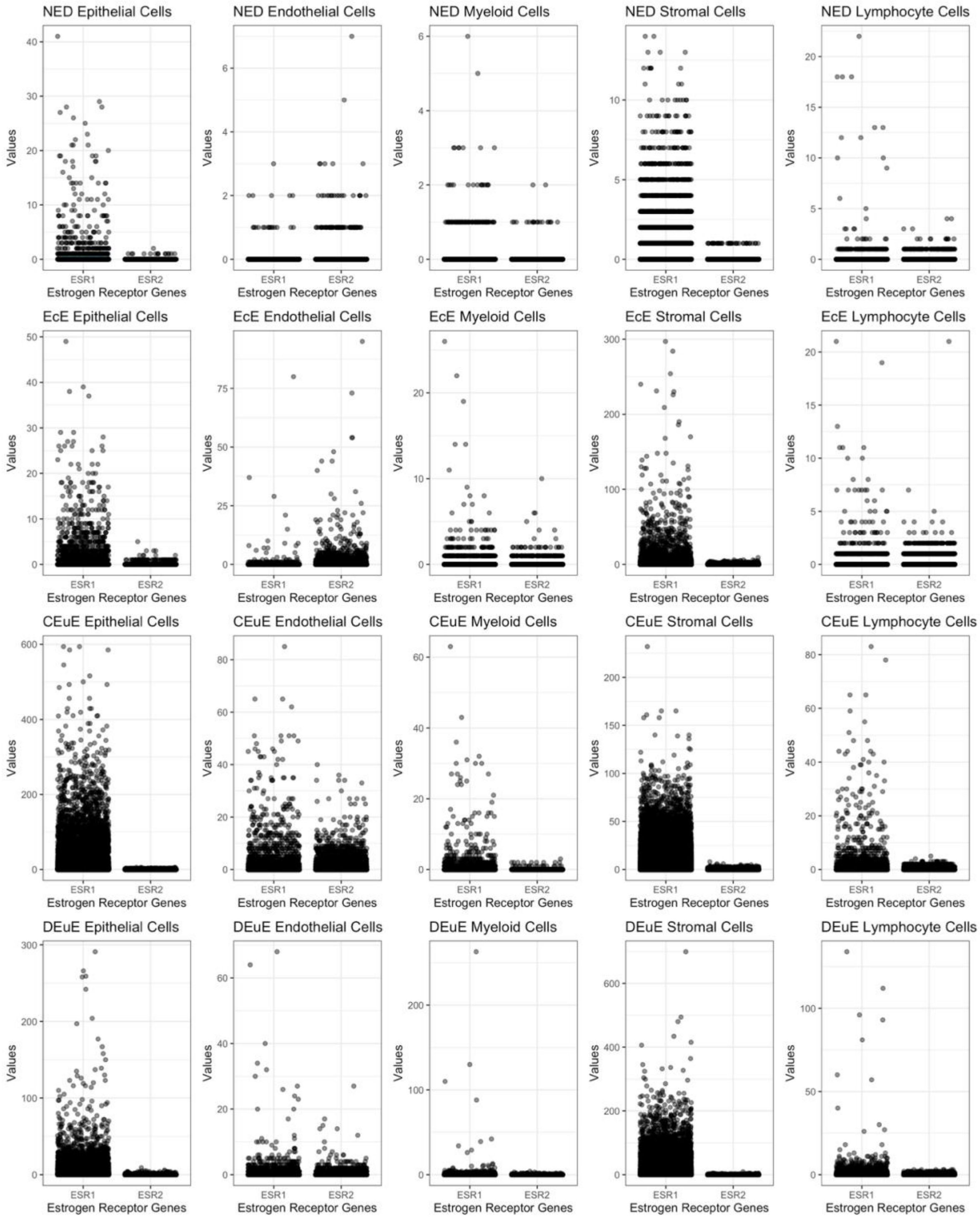

Supplementary Figure 1: Each boxplot shows the distribution of raw counts for either ESR1 or ESR2. Each panel represents a specific tissue and cell type subset – each row also represents one tissue while each column represents a cell type. ESR1 visually is expressed higher than ESR2 in general.

bioRxiv preprint doi: https://doi.org/10.1101/2025.09.15.676330; this version posted September 18, 2025. The copyright holder for this preprint (which was not certified by peer review) is the author/funder, who has granted bioRxiv a license to display the preprint in perpetuity. It is made available under aCC-BY 4.0 International license.

Supplementary Figure 2

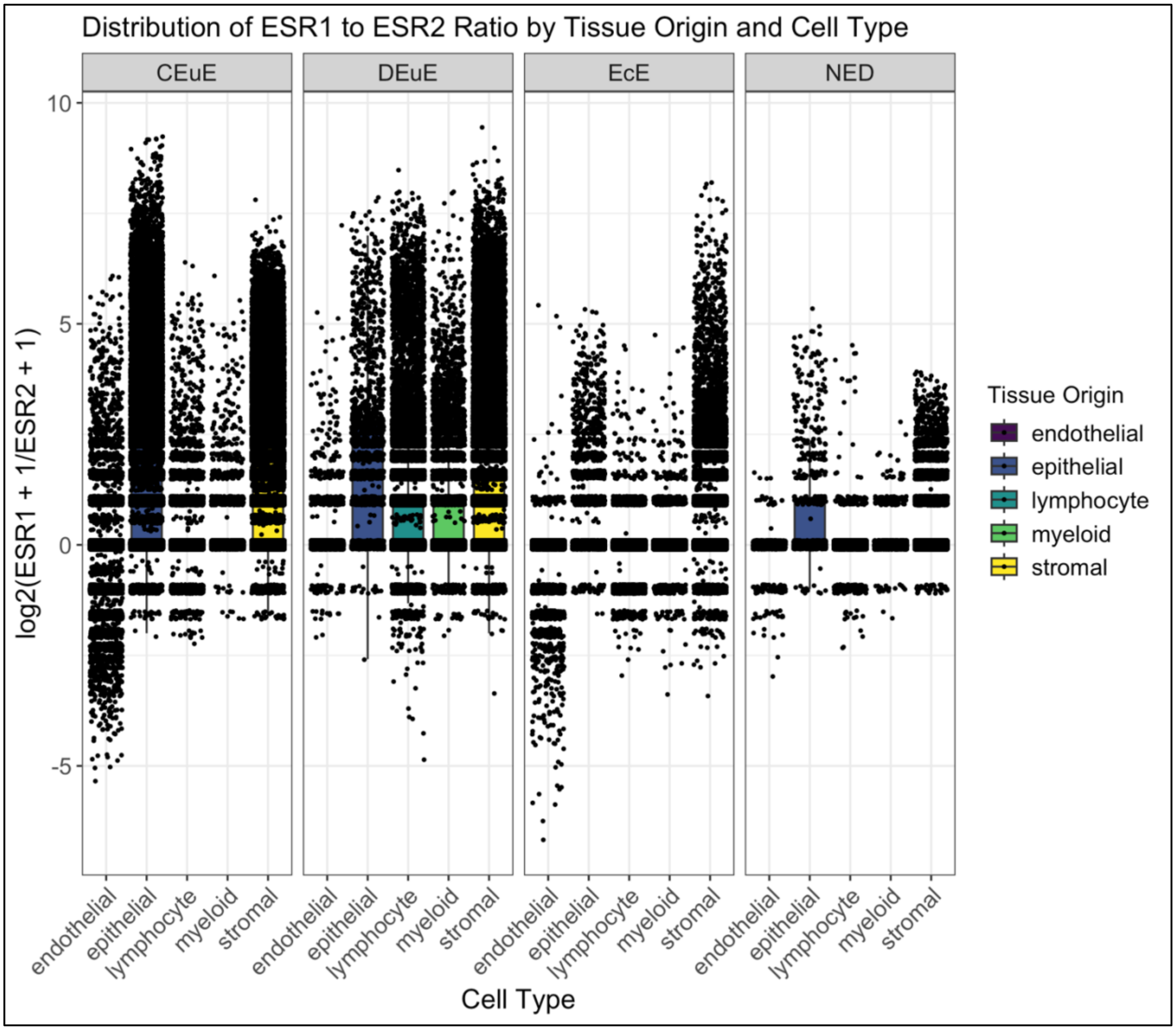

Supplementary Figure 2: These boxplots are split into four panels, containing one panel for each tissue type – Control Eutopic Endometrium (CEuE), Disease Eutopic Endometrium (DEuE), Ectopic Lesions (EcE), Disease-Free Peritoneum (NED). In each panel, there is a boxplot for each identified cell type containing a point for every cell's log2(ESR1/ESR2) in that subgroup. When comparing distributions of these ratios using Wilcoxon Rank Sum between cell types in each tissue origin separately, every comparison was significant (p value <0.05) except for one. In CEuE tissue, the epithelial to stromal comparison was insignificant, indicating the distribution of these ratios were not significantly different from one another.

bioRxiv preprint doi: https://doi.org/10.1101/2025.09.15.676330; this version posted September 18, 2025. The copyright holder for this preprint (which was not certified by peer review) is the author/funder, who has granted bioRxiv a license to display the preprint in perpetuity. It is made available under aCC-BY 4.0 International license.

Supplementary Figure 3

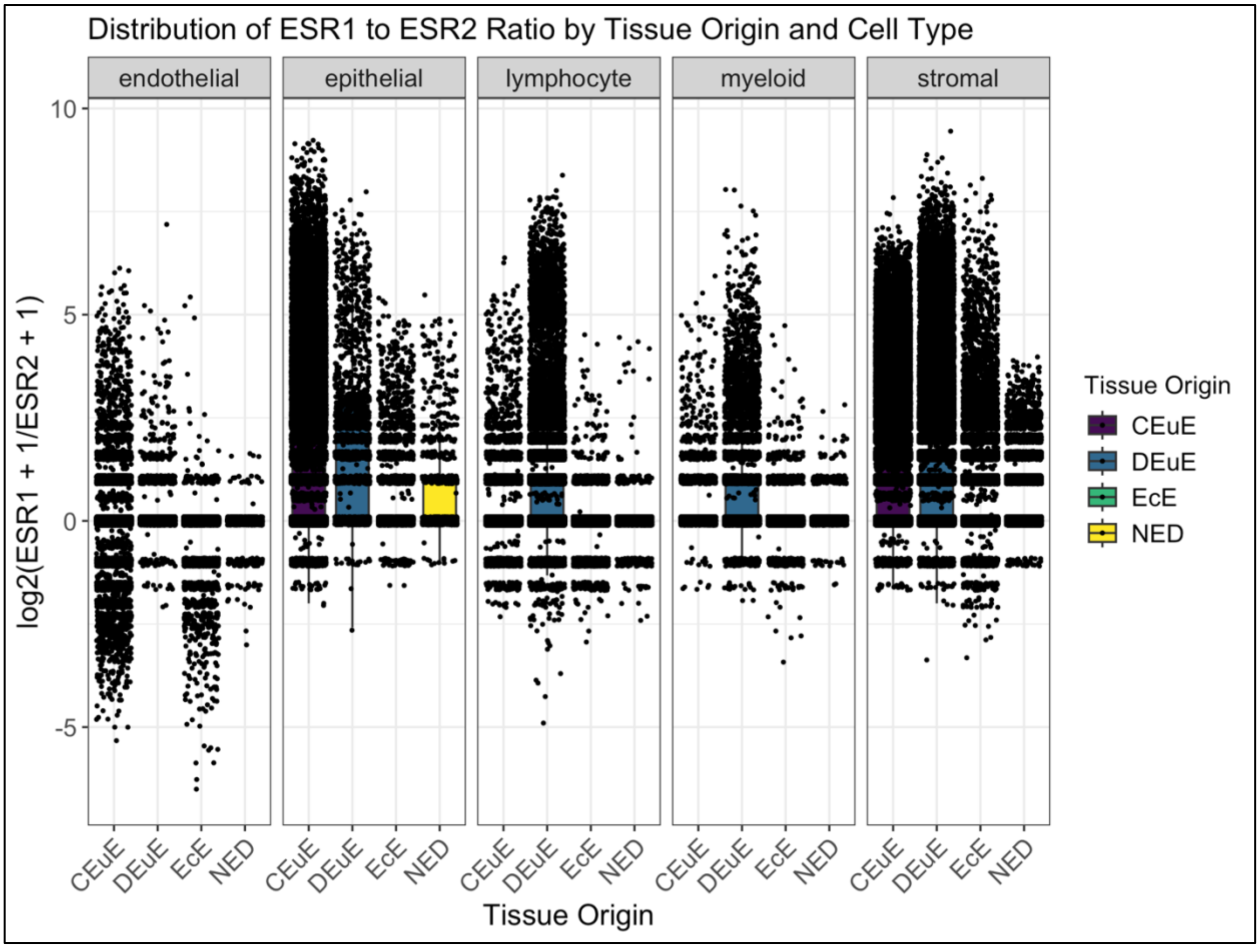

Supplementary Figure 3: These boxplots are split into five panels, containing one panel for each cell type – endothelial, epithelial, lymphocyte, myeloid, and stromal. In each panel, there is a boxplot for each tissue type – Control Eutopic Endometrium (CEuE), Disease Eutopic Endometrium (DEuE), Ectopic Lesions (EcE), Disease-Free Peritoneum (NED) – containing a point for every cell's log2(ESR1/ESR2) in that subgroup. When comparing distributions of these ratios using Wilcoxon Rank Sum between tissue origins in each cell type separately, every comparison was significant (p value <0.05) except for two. NED to EcE lymphocyte cells (p value = 0.2) and CEuE to NED endothelial cells (p value = 1).

bioRxiv preprint doi: https://doi.org/10.1101/2025.09.15.676330; this version posted September 18, 2025. The copyright holder for this preprint (which was not certified by peer review) is the author/funder, who has granted bioRxiv a license to display the preprint in perpetuity. It is made available under aCC-BY 4.0 International license.

Supplementary Figure 4

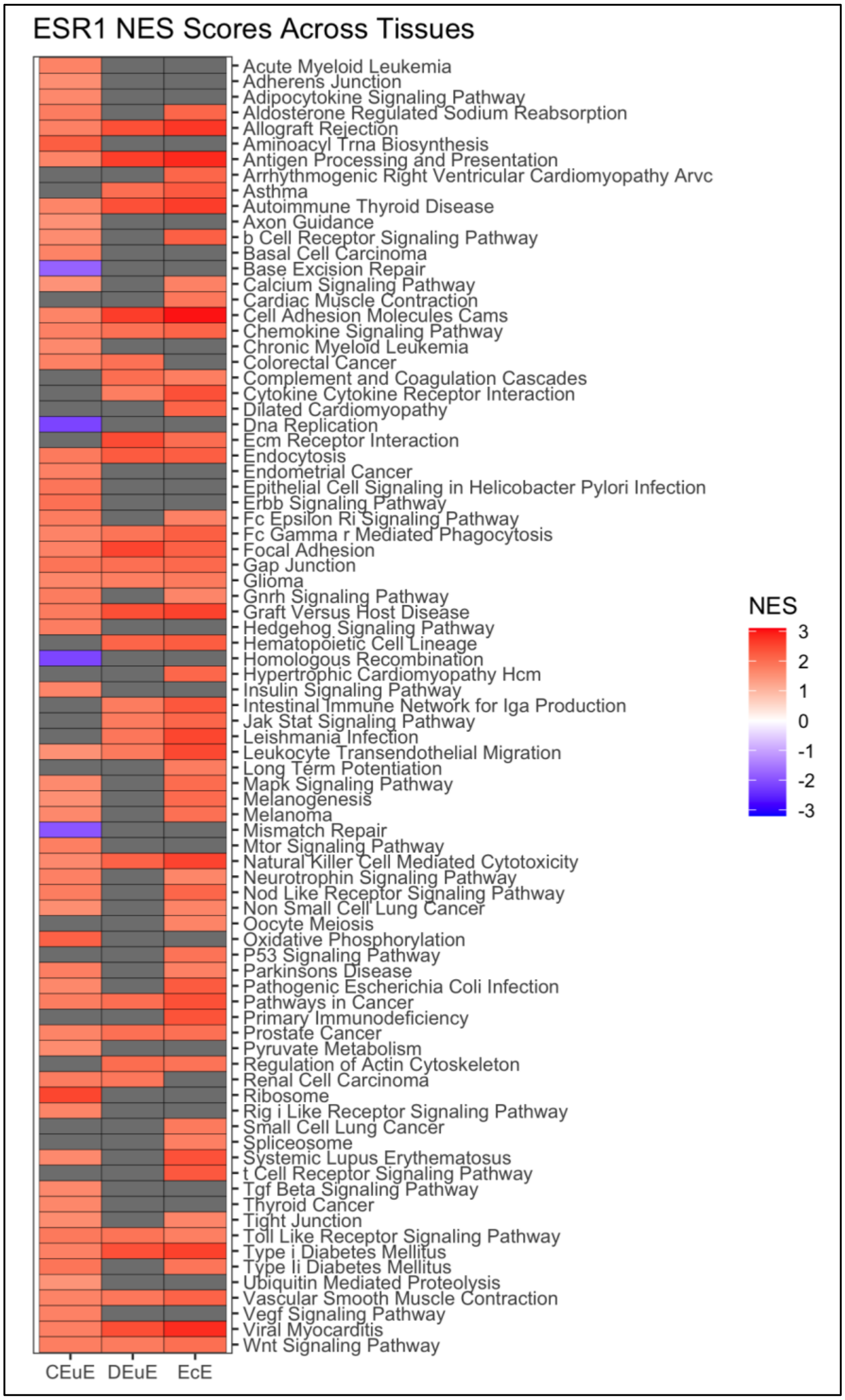

Supplementary Figure 4: This heatmap compares the normalized enrichment scores of all enriched pathways with adjusted p values less than 0.01 from ESR1+ cells compared to null expressing cells across three tissue types: Control Eutopic Endometrium (CEuE), Disease Eutopic Endometrium (DEuE), and Ectopic Lesions (EcE). The gradient ranges from blue to red with a darker blue indicating a more negative normalized enrichment score and darker red indicating a more positive normalized enrichment score.

bioRxiv preprint doi: https://doi.org/10.1101/2025.09.15.676330; this version posted September 18, 2025. The copyright holder for this preprint (which was not certified by peer review) is the author/funder, who has granted bioRxiv a license to display the preprint in perpetuity. It is made available under aCC-BY 4.0 International license.

Supplementary Figure 5

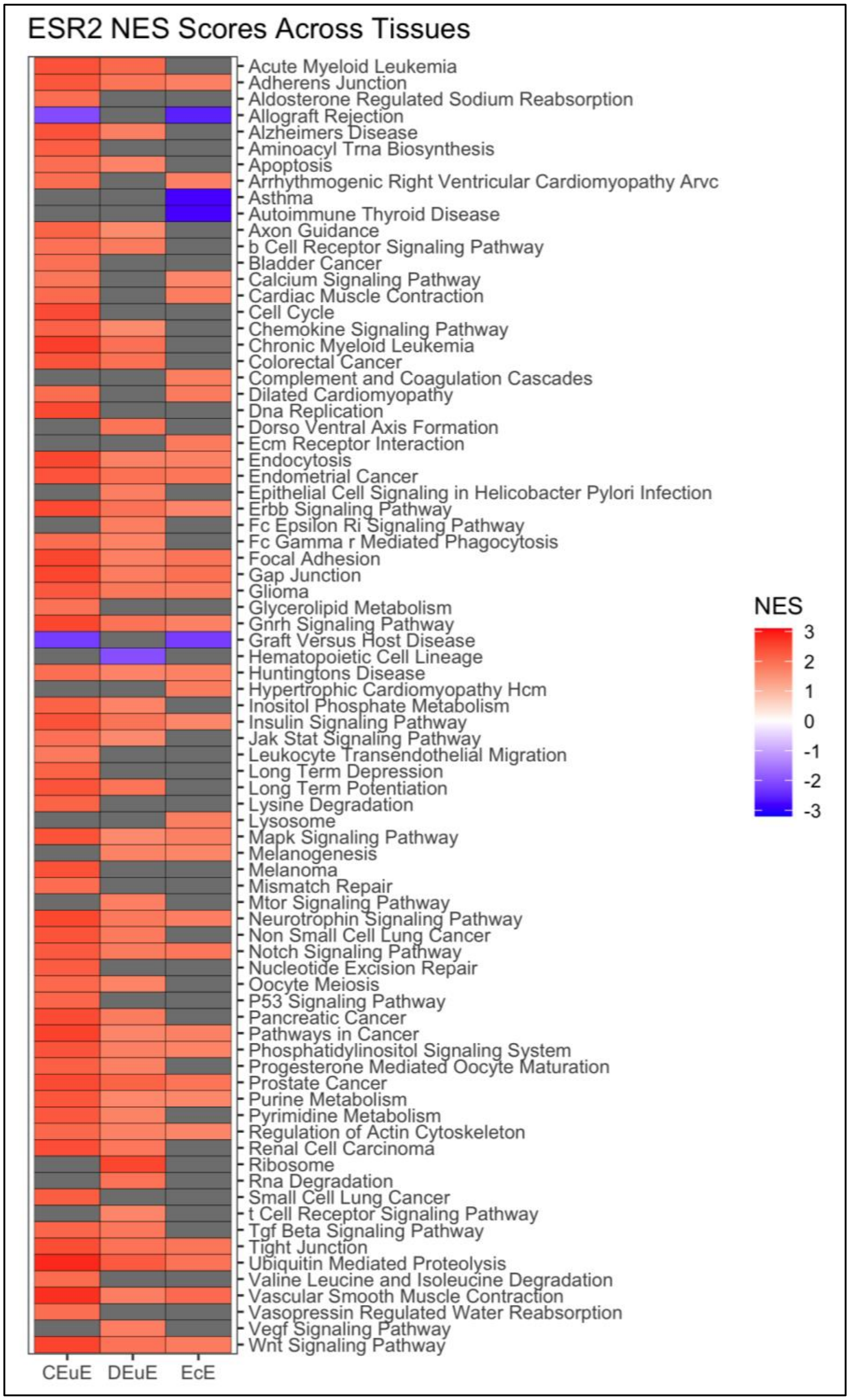

Supplementary Figure 5: This heatmap compares the normalized enrichment scores of all enriched pathways with adjusted p values less than 0.01 from ESR2+ cells compared to null expressing cells across three tissue types: Control Eutopic Endometrium (CEuE), Disease Eutopic Endometrium (DEuE), and Ectopic Lesions (EcE). The gradient ranges from blue to red with a darker blue indicating a more negative normalized enrichment score and darker red indicating a more positive normalized enrichment score.

bioRxiv preprint doi: https://doi.org/10.1101/2025.09.15.676330; this version posted September 18, 2025. The copyright holder for this preprint (which was not certified by peer review) is the author/funder, who has granted bioRxiv a license to display the preprint in perpetuity. It is made available under aCC-BY 4.0 International license.

Supplementary Figure 6

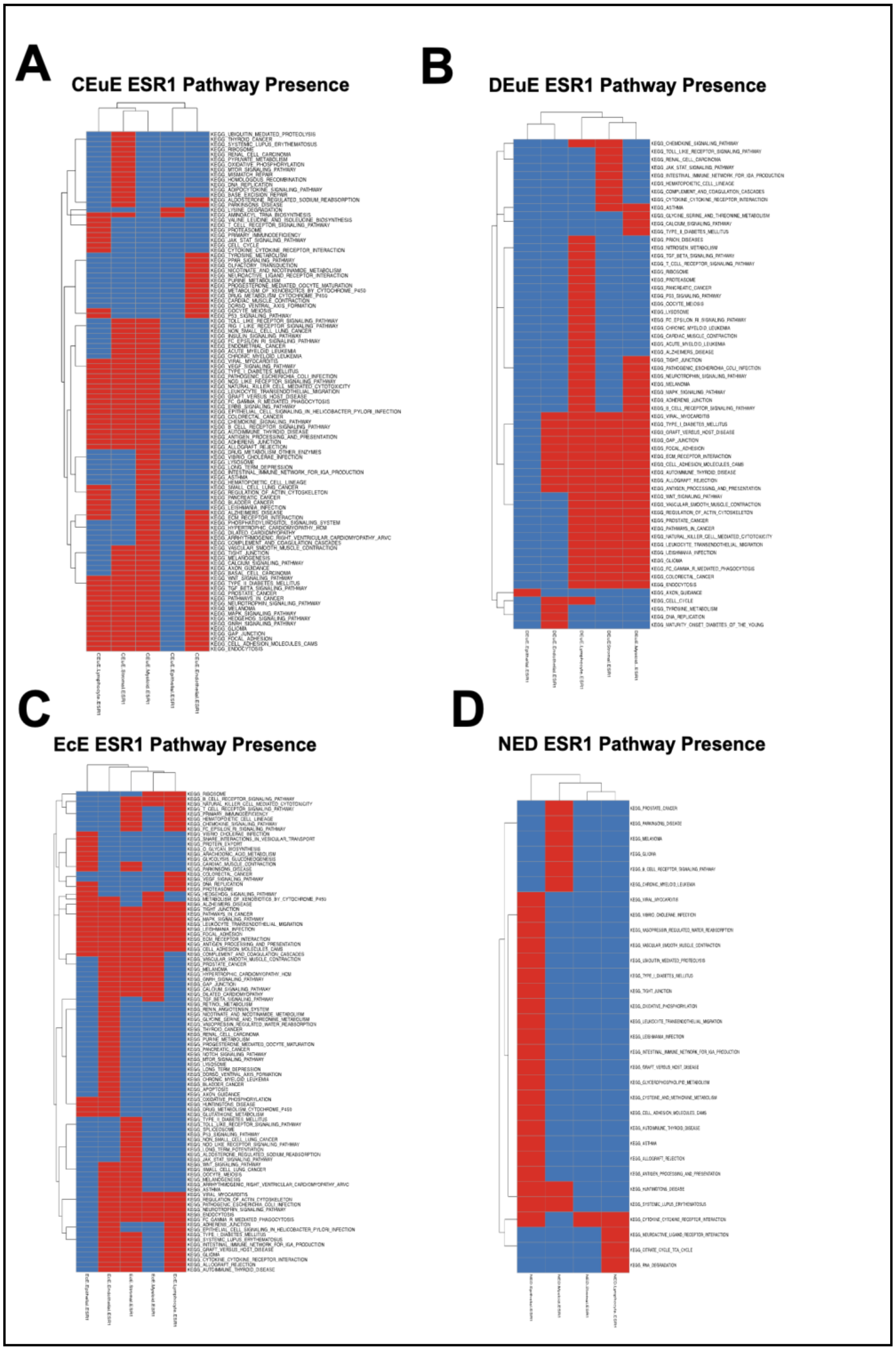

Supplementary Figure 6: Pathway Presence across ESR1+ cell types in each tissue origin of interest. Red color indicates the pathway is significantly enriched in that subgroup. Blue color indicates the pathway is not significantly enriched in that subgroup.

bioRxiv preprint doi: https://doi.org/10.1101/2025.09.15.676330; this version posted September 18, 2025. The copyright holder for this preprint (which was not certified by peer review) is the author/funder, who has granted bioRxiv a license to display the preprint in perpetuity. It is made available under aCC-BY 4.0 International license.

Supplementary Figure 7

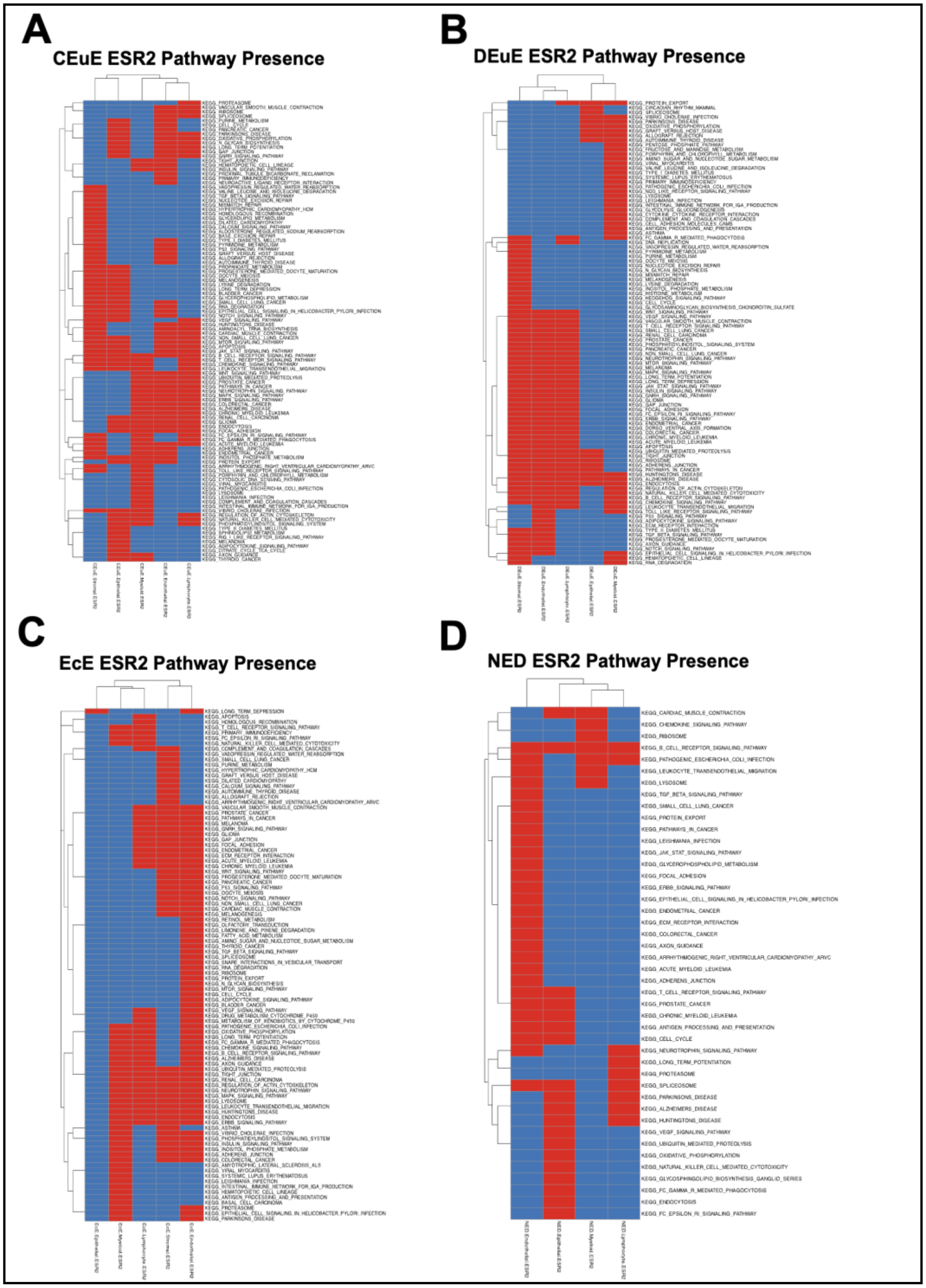

Supplementary Figure 7: Pathway Presence across ESR2+ cell types in each tissue origin of interest. Red color indicates the pathway is significantly enriched in that subgroup. Blue color indicates the pathway is not significantly enriched in that subgroup.
